# Pre-exposure prophylaxis (PrEP) in an era of stalled HIV prevention: Can it change the game?

**DOI:** 10.1186/s12977-018-0408-3

**Published:** 2018-04-02

**Authors:** Robyn Eakle, Francois Venter, Helen Rees

**Affiliations:** 1 Wits Reproductive Health and HIV Institute, Faculty of Health Sciences, University of the Witwatersrand, Hillbrow Health Precinct, 22 Esselen Street, Hillbrow, Johannesburg, 2001 South Africa; 2 Faculty of Public Health and Policy, London School of Hygiene and Tropical Medicine, London, United Kingdom

**Keywords:** HIV prevention, Biomedical prevention products, Pre-exposure prophylaxis (PrEP)

## Abstract

Pre-exposure prophylaxis (PrEP) for HIV prevention has evolved significantly over the years where clinical trials have now demonstrated the efficacy of oral PrEP, and the field is scaling-up implementation. The WHO and UNAIDS have made PrEP implementation a priority for populations at highest risk, and several countries have developed guidelines and national plans accordingly, largely based on evidence generated by demonstration projects. PrEP presents the opportunity to change the face of HIV prevention by offering a new option for protection against HIV and disrupting current HIV prevention systems. Nevertheless, as with all new technologies, both practical and social requirements for implementation must be taken into account if there is to be sustained and widespread adoption, which will also apply to forthcoming prevention technologies. Defining and building success for PrEP within the scope of scale-up requires careful consideration. This review summarises where the PrEP field is today, lessons learned from the past, the philosophy and practicalities of how successful programming may be defined, and provides perspectives of costs and affordability. We argue that a successful PrEP programme is about effective intervention integration and ultimately keeping people HIV negative.

## Introduction

Pre-exposure prophylaxis (PrEP) for HIV prevention has evolved significantly since the early conceptualization of protection tested in animal models [1] following evidence of prevention using antiretrovirals for occupational and non-occupational post-exposure prophylaxis [2, 3]. Since then, clinical trials have demonstrated the efficacy of oral PrEP, with evidence from 18 studies showing that “PrEP significantly reduced the risk of HIV acquisition” [4]. However, the level of efficacy varied according to differences in adherence within and across the study populations, with MSM showing higher levels of efficacy than found in the women-only studies [4]. Adherence is a central component for consideration of programme planning, budget, and PrEP effectiveness.

The primacy of a combined effort including early antiretroviral treatment (ART) for HIV-positive people, rendering them non-infectious, and efficient prevention interventions for HIV-negative people including condom distribution, treatment of sexually transmitted infections (STIs), post-exposure prophylaxis, and oral PrEP, voluntary medical male circumcision (VMMC), as well as continued outreach and education programming, is necessary if the goal of controlling the epidemic by 2030 is to be realized [5]. The World Health Organization (WHO) and UNAIDS have made PrEP implementation a priority for populations at “substantial risk” [6, 7], and several countries have developed guidelines and national plans integrating PrEP into programming, with the United States, South Africa, and Kenya among the first with official government-supported guidance [8–12]. These guidelines have been developed based on evidence emerging from demonstration projects [13].

Developing relevant and successful PrEP interventions, as well as defining what success is for those programmes is a challenge that the HIV prevention world is currently evaluating and debating. Since PrEP will not be a standalone intervention and rather integrated into existing programming and systems, measures of success should take into account combination prevention and programming as a whole. In addition, data suggest that while men can achieve good protection with PrEP even if doses are missed, women need to take PrEP every day to achieve high levels of efficacy [14, 15]. Evaluating effectiveness in a female population is thus strongly influenced by adherence, making its performance in the field harder to predict.

Mathematical modelling has suggested that PrEP could be part of changing the HIV prevention game, with the potential to enhance conventional prevention efforts, depending on the ability of programmes to prioritise those at risk and manage costs [16–19]. There is a desperate need for improved prevention efforts, so understanding how to strategically focus PrEP interventions to achieve optimal outcomes and to reinvigorate prevention programmes, is of critical importance. Coupled with strategic planning is the need for demand and support for the intervention. In the United Kingdom (UK), grass-roots advocacy and support largely from the men who have sex with men (MSM) community has pushed for expanded availability of PrEP beyond those able and willing to pay out of their own pockets [20]. In Swaziland, a national pilot is underway to capitalize the success of the implementation of test and treat for HIV-positive people by adding PrEP as an option for those at higher risk of HIV in the general community [21]. These are just two separate examples of where ground up and top down support have pushed the availability of PrEP into a position to make a difference as a prevention system disrupter.

This review summarises the literature on where the PrEP field is today, discusses lessons learned thus far from intervention and service delivery integration salient to the introduction of PrEP, discusses the philosophy and practicalities of how successful PrEP programming may be defined, explores how the newness of PrEP may be leveraged as a system disrupter to encourage demand, and provides perspectives of prevention costs and cost effectiveness. We argue that developing and measuring a successful PrEP programme is about effective prevention intervention integration aimed at keeping people HIV negative.

## PrEP: Where are we now?

Oral PrEP is now included as part of the recommended standard of prevention by the World Health Organization (WHO) for people defined as being at “substantial risk” of HIV infection [6]. Substantial risk was defined in the 2016 Consolidated Guidelines on the Use of Antiretroviral drugs for Treating and Preventing HIV as geographical incidence of 3% or higher. However, the recommendation also suggests that 2% is sufficient, and considerations of population context, as well as demand should be taken into account, thereby effectively allowing countries to interpret this definition as it is relevant to their particular settings. Oral PrEP has been registered for use by sexually active men and women, by several national drug regulatory authorities including the United States Food and Drug Administration (US FDA) and the South African Medicines Control Council (MCC). Implementation studies for different target populations have been completed or are in various stages across the globe [13].

The current oral PrEP strategy itself requires a daily commitment to pill taking, in particular for heterosexual women who appear to require higher concentrations of antiretrovirals in the genital tract to confer protection [14]. This is supported by preliminary evidence which suggests that in order to reach adequate drug levels in tissues exposed to potential HIV infection (e.g. vaginal and/or anal), women require near perfect adherence to a seven day regimen, where MSM may reach adequate levels in anal tissues with only 4 days of pill taking in a week which can be non consecutive [14, 15]. Because of the significant behaviour requirements to maintain consistent daily pill taking, additional options for PrEP delivery, including long-acting injectables, vaginal rings and films, are being developed to increase the selection of PrEP products and allow people to make choices about which technology best fits their lifestyles [22–24]. This scope of development is comparable to contraception where increasing the number of contraceptive options has been shown to significantly increase the overall uptake of contraception [25]. This seems to also be the case with the female condom where expanding the choice of product has improved access and use [26]. A vaginal ring containing the HIV drug dapivirine was recently tested in clinical trials and showed modest efficacy in preventing HIV, and an open label trial is now ongoing [27, 28]. Other products are in development, and it may take a few years to complete clinical efficacy studies, secure licensure and assess needs for implementation and cost [29]. In the meantime, noting the persistently high HIV incidence particularly among adolescent girls and young women (AGYW), and other vulnerable populations, it is critical to rollout oral PrEP as an expansion of prevention technology options and learn from the experiences of scaling it up which can later ease the way for new products.

**Fig. 1:**
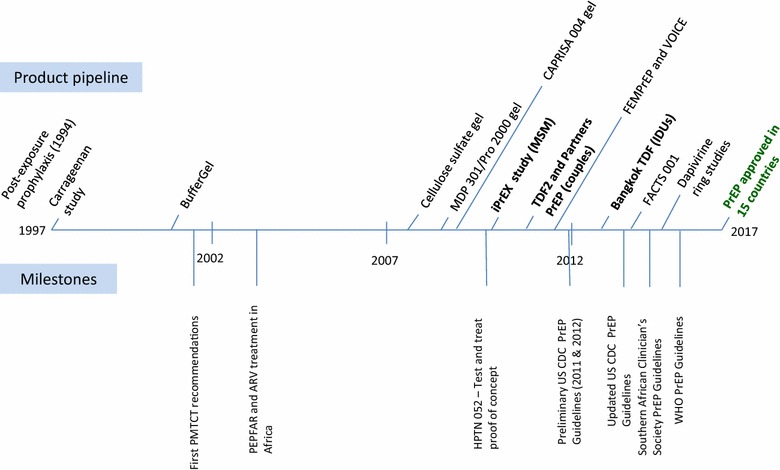
An illustration of the last 20 year of ARV-based prevention product development. Events above the timeline are related to the ARV-based prevention product pipeline and those below the line denote important milestones related to HIV prevention and care over the past 20 years. Dates and products represent timing of results unless otherwise noted. Data for this timeline were extracted from several sources [30–34]

Figure 1 shows a timeline for the last 20 years of ARV-based prevention development and demonstrates the rapid increase in development activities over the last 10 years compared with the previous decade. This timeline depicts the ‘scale-up’ in knowledge which happened over a period of time, gaining momentum with the results of each product. Although not all of the products or stepping stones illustrated here are of equal impact, each step in the development process contributed to the evolution of thinking and decisions in the development of ARV-based prevention technologies. Now, as the oral PrEP intervention is scaled-up in countries and new products come online, a similar escalation in learning from and accelerating implementation will ideally occur. Additional graphics and details of development can be accessed on the AVAC website [30].

 Per recommendations from mathematical modelling studies [16, 18, 35] as well as the WHO [36], first wave implementation studies have focused on delivering PrEP to populations at higher risk of HIV, in particular MSM and sex workers, as well as people who use drugs (PWUD) and AGYW to a lesser extent. This approach was aimed at optimising impact and cost effectiveness. In practice, these projects have mostly focused on MSM and sex workers where programmes have already been established as a sort of “low hanging fruit” [13]. Beyond these first waves in defined programmes, however, identifying those at highest risk and prioritising risk groups brings significant challenges. Most populations at highest risk experience structural vulnerabilities such as lack of access to services, lack of information and/or criminalization making them harder to reach [37]. Others have pursued studies for the harder to reach such as such as the SAPPH-IRe trial in Zimbabwe which combined provision of PrEP and test and treat through mobile clinics in rural areas throughout the country [38]. Second wave projects have been focusing on AGYW, which, while comprising only 11% of the population, are estimated to make up 20% of new HIV infections globally and are in great need of additional HIV prevention options [7, 39]. However, beyond sexual and reproductive health services, to varying degrees depending on settings, AGYW are more difficult to engage in care or interventions such as PrEP because they are not necessarily grouping together in particular clinics or geographical/contextual areas (as opposed to MSM or sex workers) and therefore require a whole different approach to communication, education, and support for PrEP use.

From the few results reported thus far from demonstration projects, it appears that people at high risk are self-identifying and taking up PrEP [40, 41], but it may be that these people represent “early adopters” as the numbers are relatively small. These early adopters sit at the front of the bell curve that represents the Diffusion of Innovations theory [42]. This theory states that uptake of any new technology or behaviour starts with a small proportion of people adopting and promoting it to their communities. Perhaps one of the greatest challenges in PrEP delivery will be to engage the next level of adopters who are at high risk, but may not initially be highly motivated to take it up. Promoting uptake, or generating demand in more sceptical communities, may involve a combination of strategies including social marketing and creative adherence support driven by lessons learned from outside the public health arena such as behavioural economics [43, 44]. Approaches will also have to be tailored according to each population and geography. In this regard, viewing PrEP itself as a system “disrupter”, where the newness and promise of the product is a motivator in and of itself, will both require new strategies to engage PrEP-sceptical potential end-users as well as those who may have become impervious to prevention messaging and disinterested in available technologies. This element of capitalizing on the introduction of a new prevention option could be an important component of generating demand among the next set of adopters.

## New prevention interventions: integration, programming, and lessons learned

The implementation of oral PrEP comprises a few critical logistical components. These include initial HIV testing to confirm an HIV-negative status, continued testing to ensure no change in HIV status, and monitoring of kidney health. These are the basic clinical requirements as recommended in several guidelines [6, 8, 11, 12]. Additionally, these guidelines highlight the need to monitor for potential hepatitis B infection, which does not exclude but could complicate PrEP use, other potential co-infections, and especially women who may be pregnant or breastfeeding where guidance on PrEP use is mixed. These components as currently stipulated require informed healthcare providers who can support their clients with information when they are making the decision as to whether to start PrEP, as well as support them during their time using PrEP with tailored adherence strategies.

With these requirements in mind, it is clear that new prevention technologies or interventions may be challenging to integrate into existing services if the specific requirements of introduction are different from what exists in the established system. Male condoms are the commonest and most successful prevention technology so far introduced. Overall they are inexpensive and widely utilised [45, 46], however, no intervention is perfect and male condoms have suffered from challenges like any other. Programmatic challenges have included issues with reliable distribution and access, as well as negative social connotations around trust and sex, and some reported problems with breakage [47–53]. Female condom programmes have been more challenging to introduce and sustain because of higher unit costs, limited distribution outlets, negative health care worker attitudes, and user acceptability [54, 55]. Similar challenges should be anticipated in the introduction of oral PrEP programme, as many female condom programmes floundered because of these obstacles. As with male and female condoms, consideration of where PrEP can and should be delivered will be critical so as not to limit access or stigmatise the product. Potential outlets could be specialised key population clinics, general public health clinics, school health programmes, and sexual and reproductive health clinics, as well as mobile versions of all of these.

Another example is VMMC, a relatively simple, one-time surgical, typically outpatient procedure, and once done offers lifelong partial protection against HIV. However, it took time to build VMMC into a viable service from both the provider and the client perspectives. From the service delivery side, factors including “country ownership; sustained political will; service delivery efficiencies, such as task shifting and task sharing; use of outreach and mobile services; disposable, pre-packaged VMMC kits; external funding; and a standardized set of indicators for VMMC” were found to be the ingredients required for successful implementation and scale-up, while continual failures in supply chain management and unreliable funding sources caused issues in maintaining consistent service provision [56]. PrEP will need some of these same components to be successfully implemented and scaled-up, and in addition will have challenges such as repeat HIV testing, blood draws, and regular client follow up. These ingredients for implementation speak to the required ownership, accountability, and pragmatism of integrating a new prevention intervention into systems already burdened with heavy patient volumes and logistical management issues.

Implementation of VMMC taught important lessons which could also inform PrEP rollout. Issues such as task shifting, the need for specific, easy to use kits for VMMC and the negative response of some men to compulsory HIV testing, had not been anticipated as challenges [57]. To address these issues, more resources were needed to develop strong community-based social marketing campaigns as well as mechanisms to support men. Resources were aimed at messaging for men and women to promote the intervention, and in some cases, providing cancer screening services for women to promote a holistic health programme [58]. The observation that adjustments to the VMMC programmes were required once implementation had begun, demonstrates the importance of continuous, iterative programme review once a new technology is introduced. As with VMMC, developing supportive partner services, or options for partner engagement, could strengthen overall PrEP services and help to mitigate stigma and rumours arising from misunderstanding of the intervention.

Additionally, for PrEP to become normalized as an intervention, communities will need to become familiar and accepting of the concept. This will be challenging if only certain key populations are prioritised for rollout [59]. Messaging around the intervention will need to consider social aspects of delivery which were also barriers in VMMC implementation. These include the potential for loss of income when at the clinic, fear of the procedure or of side effects in the case of PrEP, lack of HIV risk perception, and lack of partner support [60–62].

Finally, and perhaps most unique to PrEP, is that it will be aimed at maintaining an HIV negative status in those at risk for HIV and will be used during periods of high risk rather than for a person’s entire life. The social and risk element is especially complex with the potential difficulties of identifying the high-risk groups, maintaining engagement with them while mitigating stigma (and criminalization for some marginalised groups), providing tailored services that are acceptable, and having the individuals who are identified as being at risk, self-assess sufficiently to ensure adequate adherence. Getting these pieces right will also depend on how the larger community understands the product (e.g. not as a “sex worker or MSM product”), and accepts and supports its implementation. The use of PrEP for limited portions of time will also add to the complexity of maintaining use during the time of need in these high-risk populations and conveying appropriate messaging to that extent.

## The focus on adherence

Like oral contraception, PrEP is a highly effective prevention technology if taken consistently, notwithstanding the different efficacy requirements for women and men mentioned above. Adherence to PrEP is therefore key to the method’s success, yet there have been many challenges in ensuring adherence in clinical trials. Adherence to PrEP (or lack thereof) was why parts of one study [63], and another entire study [64] were not able to adequately measure efficacy among women, where at least 70% of participants in the VOICE trial and 60% in FEMPrEP did not use PrEP properly. The qualitative research published following the VOICE [65] and FEMPrEP [66] studies revealed highly nuanced reasons for lack of use, including misconceptions about personal risk, logistical issues attending the clinic, apathy towards research, and general lack of interest in the product but intense interest in the high quality health services provided by the clinical trial clinics [65, 66]. This presented a challenge in the trials where women would tell clinical staff that they were adhering to their assigned regimens in order to keep coming to the clinic. Some observers questioned whether oral PrEP would ever be a viable product for women who, in large numbers in these studies, demonstrated little interest in consistent PrEP use. However it was generally agreed that in the absence of many prevention choices, that the product should be made available following additional research into the nuanced feedback received from the trials and previous prevention efforts [43, 67–70].

These insights are important as they point towards a need to conceptualize oral PrEP differently from other prevention methods and from the provision of ART, as well as build PrEP interventions into care valued by the community. Importantly, the duration of use will be determined by the needs of the individual. Taking PrEP over a period of time has been likened to “seasons of risk”, where someone may choose to take PrEP for a time, and then switch to another method [43, 71], as happens with contraception. As with contraception, as long as this engagement with products results in the desired outcome (maintaining a negative HIV status), then the programming will be successful—e.g. PrEP does not have to be implemented in place of or to the detriment of male and female condoms, or other prevention strategies.

The provision of ART, especially as it expands into test and treat programming worldwide, has focused on uptake and consistent use of HIV treatment for life. This cannot be the mind set for PrEP, which must be delivered by providers with a message of flexibility thus promoting honest feedback from clients who will need to engage with the best product available to stay negative. Presenting PrEP to health care workers as being analogous to contraception is likely to be better understood rather than locating PrEP alongside HIV treatment. In addition, the early experiences of ART and its side effects persist in the memories of communities, which could be off putting for clients considering PrEP. With these issues in mind, supportive adherence counselling will be imperative for PrEP success [72].

## Defining and building success

The primary measure of success for any HIV prevention technology is the number of HIV infections averted over a period of time and in a prescribed population. However, this retrospective analysis can take a significant effort and time to produce. To more accurately assess progress in shorter periods of time (quarterly or even annually), measurement of success is often limited to programmatic counting. For instance, the number of condoms distributed to a given population in a given year is usually taken as whether a condom campaign has been successful. This metric, however, does not shed light as to whether people used the condoms.

Overall, for evaluating PrEP success, numbers of HIV infections averted will need to be modelled based on a range of composite programmatic measures. One important metric will be uptake among those eligible for PrEP. However, eligibility will have to be well-defined and is currently different in some countries. There is clinical eligibility of being HIV-negative with healthy kidney function which is universal in guidelines, however the question of risk is where countries currently differ. Kenya is prioritising people at higher risk in certain geographical areas [73], while South Africa is focusing only on key populations and has excluded pregnant and breastfeeding women for the time being [10, 12]. Therefore, any comparisons of uptake across countries will need to keep these differences in mind. In addition, the denominator for who is actually eligible may be difficult to calculate depending on the validity of population demographics in a given setting.

Measuring uptake should also go a step further in determining the number of people newly engaging in or returning to care because of interest in PrEP. Since PrEP may actually act as a catalyst to reignite interest in HIV prevention services overall then accounting for increases in numbers of people coming to the clinic because of PrEP, whether they end up using it or not, is also an important measure of success for the larger prevention goal of staying HIV-negative. This will depend, however, on programmes having reliable data from before PrEP is introduced to be able to assess increases in numbers, as well as ideally recording in a standardized manner the purpose for engagement in care as well as date of previous HIV test and/or visit. In addition, it will be important to then analyse who is coming to the clinic in terms of (on a basic level) age, sexual behaviour, and gender.

Retention is a critical metric, but is also not so straight forward as there is the question of whether it is retention with use of PrEP, or rather maintaining consistent retention in care. Arguably, the latter could pose a better metric provided individuals remain HIV-negative given that is the primary purpose of PrEP and engagement in prevention services in the larger picture. Potentially both should be included, but in either case, the number of people returning for services will be important to capture. This brings a whole host of issues around how to track health service clients which are being tackled and tested through multiple creative efforts such as health cards, biometrics, and/or national electronic databases. The retention aspect should also take into account the notion that some people may fall out of PrEP use and so should be able to track whether they maintain consistent engagement with the system while they are in periods of lesser HIV risk [72, 74].

For the metric of retention including PrEP, there has been evidence to show that recording repeat refills can be useful as a composite measure for use and one that is far more sustainably collected as compared with MEMS or pill counting [72]. As with condoms, just counting the number of people taking up PrEP or pills distributed will not account for whether people actually used the product. Adherence was a common metric in the PrEP efficacy studies, but the measurement required drug level analyses, pill counting and some more advanced technologies such as medical event monitoring systems (MEMS) on pill bottles to electronically record when bottles are opened. These strategies were also used to determine how reliable participants’ self-reports were. Adherence measured in these relatively sophisticated fashions is not likely to be sustainable in a real-world environment due to labour burden, facility and budget capacities. In addition, measurement of adherence needs to be nuanced considering the cycles of risk, and differing levels of efficacy between men and women.

Seroconversions to HIV-positive status can also be tracked to assess programme success in promoting and supporting PrEP use, or at the very least, success in engaging people in effective HIV prevention. Additionally, if PrEP cycling is not managed well, there will be a risk for generating ARV resistance, although the PrEP efficacy and implementation studies, as well as pharmacovigiliance research to date have shown the probability of resistance generated by PrEP to be very low [4, 75].

These indicators will inherently depend, however, on ongoing successful personal and provider assessment of risk which also does not negatively stigmatize those at higher risk. Many pilot studies are investigating the use of risk assessment tools with varying degrees of depth. Results over the coming months from these studies will provide insight as to whether these tools have been useful and to what degree they should be used in scale-up.

Finally, for there to be PrEP interventions to assess, there must be a market and therefore demand and support for them. Testing implementation in the field and paths to guidelines in the few countries which have already taken it on board have varied greatly according to context. In the UK, the PROUD study definitively demonstrated the HIV protection potential of PrEP even before the end of the trial [76] and sparked grassroots demand from the MSM population for it to be offered through the National Health System (NHS). Negotiations are still ongoing due to the cost of integrating the drug into the NHS, however analyses have shown that existing PrEP provision through private clinics has likely contributed to the significant decrease in new infections in the UK [20, 77], further motivating continuous calls for PrEP availability. In Swaziland, where the test and treat strategy for ART was taken on board and had significant effects on reduction of community viral load, the government seized the opportunity to adopt PrEP through a national pilot in order to test the most effective way to utilize this new tool to further drive down the epidemic [21, 78]. These are two examples of how PrEP is making its way into systems from the bottom up and top down, where people have seen a need and seek to implement PrEP in a strategic way. This support is instrumental in paving the way for successful programming.

## Cost-effectiveness and affordability

Since before the clinical trials reported results of PrEP efficacy, there were significant modelling efforts to estimate impact and cost-effectiveness of PrEP [79–81]. These studies relied heavily on estimated service costs as well as cost of the PrEP drugs themselves as there were no practical data from implementation. Since then, commodity costs have evolved, with tiered pricing which allow low and middle income countries to pay less for drugs than richer countries. The markets are now with opening further the availability of generic options [82].

Models have also evolved, updated and informed by efficacy estimates and service delivery costing demonstrated in rollout studies [41, 83]. Cost-effectiveness, however, will depend on the ability of programmes to efficiently integrate PrEP into existing services, and generate demand appropriately and relevantly among people at highest risk to take it up. To add to the complexity, it will also be important to consider whether PrEP become less cost-effective over time due to saturation and decreasing burden of new HIV infections necessitating the addition or increase of scale of other interventions.

In light of these complexities, an on-going challenge for many countries is how to incorporate PrEP into national plans with tight budget already allocated to existing services, such as South Africa where PrEP is being considered within the context of an ever-growing national health budget and one of the largest ART programmes in the world [83]. In Kenya, a framework for PrEP implementation was developed highlighting a projected 5-year cost for PrEP sitting at just over at $328 million, and a funding gap of about $314 million. This is based on mathematical modelling of sub-county incidence rates with population estimates aiming to geographically prioritize those at highest risk. The majority of the budget is devoted to commodity costs, knowing that the intervention is being integrated into existing services. With time, these services should adjust to make PrEP delivery more efficient and leverage the cyclical nature of individual PrEP use.

For now, no global funding programmes are providing country-level support specifically for PrEP. The United States Agency for International Development (USAID) provides some funding for PrEP through special large programme grants with specific aims. As yet, the Global Fund has only recently included PrEP in its country in its 2017–2022 strategy, but as stated it will be for select countries and it is not clear yet when this will be effected. For now, countries are making due with leveraging anything available in their existing budgets as well as special programming to get PrEP provision off the ground.

In the meantime, the recent FDA approval [82], as well as approvals in other countries such as India and South Africa, of generic PrEP drugs should help to promote the availability of lower cost drugs especially in developing country settings. Additionally, the Medicines Patent Pool, an United Nations initiative launched in 2010 with a public health business model aimed at lowering prices for essential medicines, has played an important role in the license for the PrEP combination of TDF/FTC [84]. Following an update in 2017, the MPP license for PrEP now includes 116 countries. These efforts should help to alleviate pressure on many national budgets, as well as expand PrEP markets where it is currently not included in national health plans. The HIV epidemic in developing countries, and in particular sub-Saharan Africa, are in great need of new options such as PrEP and reduction in commodity costs is imperative to making new options available a reality. Additionally, there are also people in developed countries who do not have access to PrEP and want it, such as in the UK, Canada, and much of Europe [85, 86]. In 2015, the average cost of brand name Truvada-based PrEP in the United States was $1700 per month [87], and has been reported to be between 500 and 850 euros per month in Europe [88], thus placing high hopes on lowering costs and promoting better uptake among key populations. Advocacy to push for reducing the cost of PrEP to be offered through national health plans or insurance will be key to increasing availability in both developing and developed settings.

## Conclusions

Oral PrEP is an effective HIV prevention intervention when taken consistently, and should be made easily available to those at high risk of HIV who are self-aware and able to make the commitment to be sufficiently adherent. Oral PrEP can pave the way for these new technologies, and the lessons learned in its implementation can be used to build stronger, more adaptable programmes. PrEP will not change the game on its own, but as a component of HIV programming has the ability to disrupt current systems and reinvigorate the HIV prevention field. The measurement of PrEP success should be reflected in the numbers of people coming for prevention services that include targeted PrEP, and ultimately in demonstrated reductions in new HIV infections. Nevertheless, as with all new technologies, there needs to be a social shift at a population level if there is to be sustained and widespread adoption, which will also apply to prevention technologies in the development pipeline.

 What is the cost of not implementing PrEP, or other new prevention options? Research has shown that ART will not reduce the epidemic enough to move towards elimination, or even control [5]. For many populations, the reliance on old prevention interventions means that the risk of acquiring HIV remains unacceptably high [89]. If the goal of PrEP and other prevention programming is to prevent new HIV infections, also reducing the escalating costs of ART as a lifelong public health intervention, then offering PrEP in the spirit of promoting choice, accessibility, flexibility, and efficiency should be the first step in paving the way for new HIV prevention interventions.
